# Enhancement of intrinsic emission from ultrathin ZnO films using Si nanopillar template

**DOI:** 10.1186/1556-276X-7-263

**Published:** 2012-05-22

**Authors:** Tun-Yuan Chiang, Ching-Liang Dai

**Affiliations:** 1 Department of Mechanical Engineering, National Chung Hsing University, Taichung, 402, Taiwan; 2 Department of Mechanical Engineering, National Chin-Yi University of Technology, Taichung, 411, Taiwan

**Keywords:** Ultrathin films, ZnO, Nanopillars, Atomic layer deposition, Photoluminescence

## Abstract

Highly efficient room-temperature ultraviolet (UV) luminescence is obtained in heterostructures consisting of 10-nm-thick ultrathin ZnO films grown on Si nanopillars fabricated using self-assembled silver nanoislands as a natural metal nanomask during a subsequent dry etching process. Atomic layer deposition was applied for depositing the ZnO films on the Si nanopillars under an ambient temperature of 200°C. Based on measurements of photoluminescence (PL), an intensive UV emission corresponding to free-exciton recombination (approximately 3.31 eV) was observed with a nearly complete suppression of the defect-associated, broad-range visible emission peak. As compared to the ZnO/Si substrate, the almost five-times-of-magnitude enhancement in the intensity of PL, which peaked around 3.31 eV in the present ultrathin ZnO/Si nanopillars, is presumably attributed to the high surface/volume ratio inherent to the Si nanopillars. This allowed considerably more amount of ZnO material to be grown on the template and led to markedly more efficient intrinsic emission.

## Background

Due to the wide application of, for instance, transistors [1,2], ultraviolet photodetectors [3], and piezoelectric transducers [4,5] in the industry, all of which need to use ZnO thin film, which is regarded as a promising material for optoelectronic applications. In particular, the wide bandgap of 3.37 eV and large exciton binding energy of 60 meV (much higher than that of ZnSe (40 meV) and GaN (25 meV)) inherent to ZnO have been demonstrated to exhibit efficient excitonic emission at room temperature. Consequently, considerable efforts have been devoted to this particular material in order to harvest efficient ultraviolet (UV) emission from ZnO [6-8]. These approaches, however, required not only the thickness of 100 nm or more for ZnO thin film, but also much more time in fabricating processes. Thus, ultrathin ZnO film (<30 nm), with its low fabrication cost and time consumption, has a great potential for manufacturing optoelectronic devices. Unfortunately, owing to the relatively small amount of emissive materials involved, light emission from the planar thin films is often very inefficient with weak intensity, and therefore, it may seriously hinder the possibility of any practical applications. For that matter, it is desirable to develop a viable approach that can harvest UV emission from ultrathin ZnO films in a more efficient fashion.

In this report, a time-saving and cost-effective technique is introduced to fabricate Si nanopillars by etching a Si substrate. An alternative method of using the self-assembled silver (Ag) nanoislands as the metal nanomask to manufacture the Si nanopillars was employed. Through the Volmer-Weber growth mode during sputtering [9], Ag nanoislands were formed. With these Ag nanoislands, Si nanopillars were subsequently produced by dry etching. In general, the Si nanopillars can be obtained by the duration of about 10 s of sputter time and 5 min of dry etching. This kind of fabrication method is thus advantageous because not only the manufacturing process was very effective - only 10 s of duration for obtaining the self-assembled Ag nanomask by sputtering, but also the fabrication cost was reduced through the lithography-free, anisotropic dry etching. In addition, the ZnO/Si nanopillar heterostructures have a high surface/volume ratio, which greatly contributes to the increase of the area of UV emission from ultrathin ZnO films.

## Methods

**Figure 1:**
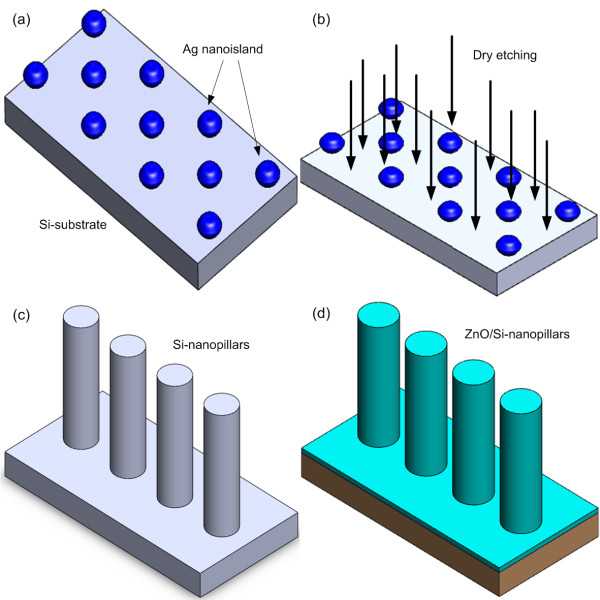
**The fabrication processes of ZnO/Si nanopillar heterostructures (not to scale).** ( **a**) Size distribution of Ag islands. ( **b**) Dry etching process. ( **c**) Formation of Si nanopillars. ( **d**) Deposition of ZnO films on the Si nanopillar surface by ALD.

Si nanopillars were produced from the self-assembled silver nanoislands which were formed via the Volmer-Weber (island growth) mode during sputtering process and subsequent dry etching process. First, the Ag nanoislands were created on Si (100) substrates by radio frequency (rf) sputtering from an Ag target for 10 s with an input power of 150 W in 25 sccm argon gas atmosphere. As shown in Figure 1a, the resulting size distribution of the obtained Ag islands is rather small, owing to the short sputtering time. In the second step, the Si nanopillar array was obtained by etching performed in a metal etcher system. Before etching, the chamber was pumped to a pressure of 3 × 10^−5^ Torr with the system temperature kept at 60°C. Subsequently, the Cl_2_ and N_2_ gases were introduced into the chamber with the flow rates of 90 and 10 sccm, respectively. After loading the prepared Si substrate which was covered with Ag nanoislands, the dry etching system was operated with the constant input power being maintained at 2,500 W and the etching time of 5 min. This dry etching process was depicted schematically in Figure 1b, in which the reactive ions passed the metal nanomask of Ag to produce the ion bombardment effect which carried out the anisotropic etching of the Si substrates to form the Si nanopillars, as shown in Figure 1c. In this practice, the Si nanopillars are about 250 to 350 nm in height with an average diameter of about 70 to 100 nm. Afterwards, the ultrathin ZnO films were deposited on the surface of the Si nanopillars by atomic layer deposition (ALD), with the ZnO film thickness estimated to be about 10 nm with 35 ALD cycles, as shown in Figure 1d. ALD is a growth technique that employs self-limiting vapor-phase chemisorption depending on consecutive surface reactions. In this work, the pulse durations of water and diethylzinc were 100 and 50 ms, respectively. The purge and pumping periods were 15 s, and N_2_ was used as the purge gas with the pressure being set at 5 × 10^−3^ Torr.

A field-emission scanning electron microscope (FESEM, JEOL JSM-6700 F, Tokyo, Japan) was used to examine the morphology of the Ag nanoislands and Si nanopillars, and energy-dispersive X-ray spectroscopy (EDS) was performed to examine the composition of the obtained samples. The structural characteristics of the ultrathin ZnO films were determined by X-ray diffraction (XRD) technique using Cu Kα radiation (*λ* = 1.54 Å) (PANalytical X'Pert Pro, Philips Inc., Almelo, The Netherlands), which was operated at 45 kV and 40 mA for grazing angle scan. Moreover, the photoluminescence (PL) was measured at room temperature using a He-Cd laser (325 nm) for excitation and a CCD with a monochromator for detection.

## Results and discussion

**Figure 2:**
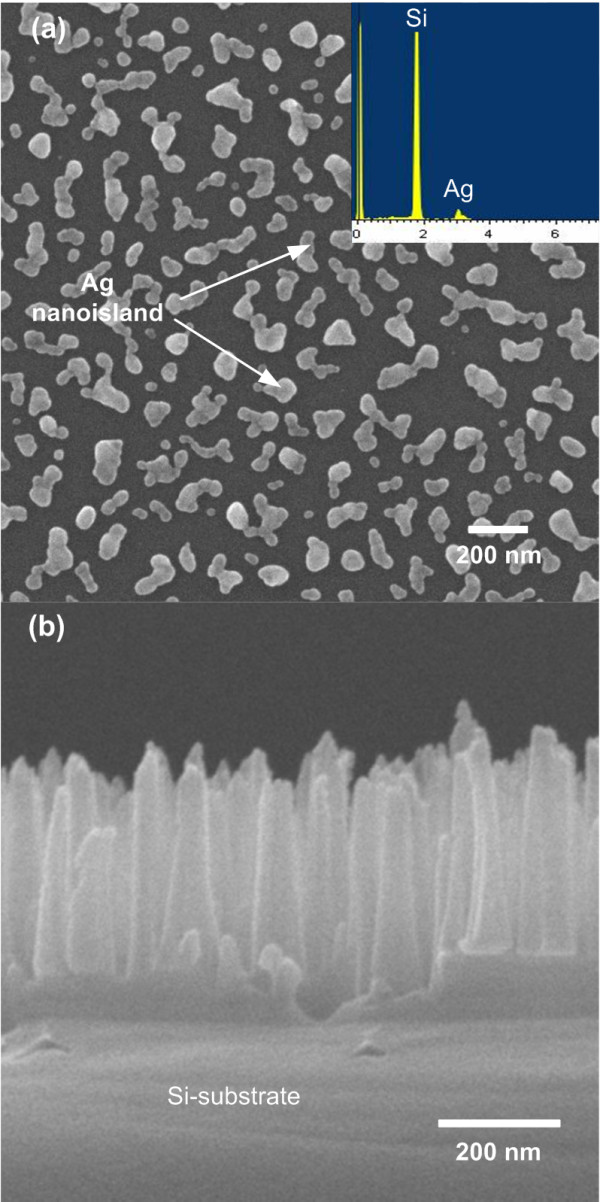
**SEM images and EDS analysis.** The SEM images of ( **a**) Ag nanoislands which were fabricated by rf sputtering and ( **b**) Si nanopillars. The inset in ( **a**) is EDS analysis of Ag nanoislands on the Si substrate.

The typical morphology and distribution of the Ag nanoislands grown on Si substrates were harvested by carrying out rf sputtering for 10 s, as shown in Figure 2a. The Ag nanomask, in this study, is created by a single sputtering step, and the Si nanopillars are created by a subsequent dry etching step. No heat treatment is needed to create these Ag nanoislands. It is important to note that the simplicity of the current fabrication method is advantageous in several respects. First, the fabrication process is very effective because it took only 10 s to obtain the self-assembled Ag nanomask by sputtering. Second, the lithography-free, anisotropic dry etching can reduce the fabrication cost significantly. The SEM image exhibits that the Ag nanoislands were isolated and small in size. In addition, the islands look like the coalescence of two or more islands from this short sputtering time. This characteristic is believed to result from the film growth mechanism of the Volmer-Weber mode [9]. Figure 2b displays the Si nanopillars obtained from the Si substrate covered with Ag nanoislands (as shown in Figure 2a) after a 5-min dry etching process. The Si nanopillars obtained under this condition are of an average height of 250 to 350 nm and are aligned vertically with an average diameter of about 70 to 100 nm. According to the result, obviously, the Ag nanoislands not only successfully served as a metal nanomask, but also effectively formed Si nanopillars by dry etching process on the Si substrate. The EDS spectrum displayed in Figure 2a for the Ag island sample does reveal the existence of Ag on the Si substrate.

**Figure 3:**
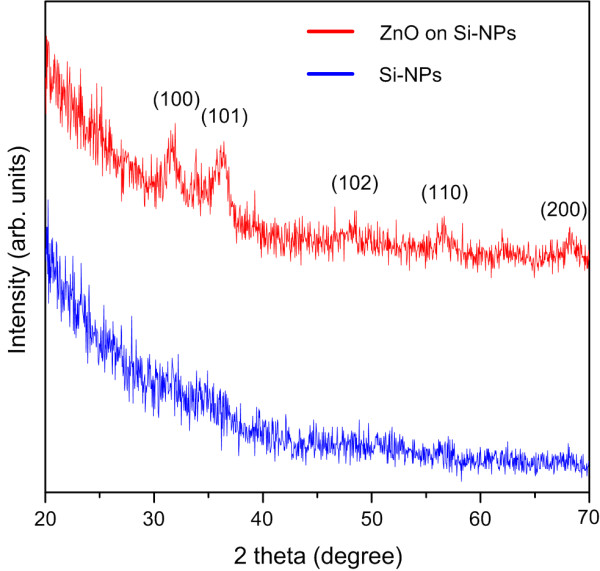
**XRD spectra.** The XRD spectra of Si nanopillars and ultrathin ZnO films which were deposited on Si nanopillars at a growth temperature of 200°C.

Figure 3 shows the XRD curves obtained for the ultrathin ZnO films deposited on Si nanopillars at a growth temperature of 200°C. It is evident that for the ZnO film grown at 200°C, there are five distinctive diffraction peaks corresponding to (100), (101), (102), (110), and (200) crystallographic orientations. The results suggest that the ultrathin ZnO film deposited on the Si nanopillars at a growth temperature of 200°C, while remaining to be largely polycrystalline, is of good polycrystalline quality.

**Figure 4:**
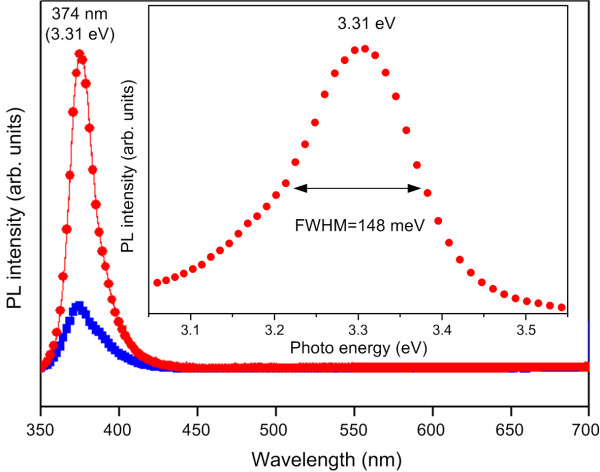
**The room-temperature PL spectra of ZnO on Si substrate and Si nanopillars.** Solid blue squares: Si substrate, solid red circles: Si nanopillars.

In order to understand the optical properties of the ultrathin ZnO films deposited on Si nanopillars at a growth temperature of 200°C, the PL spectra of the ultrathin ZnO films were examined, and the results are shown in Figure 4. Recently, Chang et al. [10] demonstrated a novel ZnO/Si nanowire template to harvest highly efficient visible emission at room temperature. In their study, the sample shows a strong visible emission for the ultrathin ZnO film deposited on Si nanowires at a growth temperature of 25°C. This result was due to the low growth temperature which made it difficult to obtain a high-crystalline quality, yet which was beneficial to a better visible emission from ZnO films. Furthermore, the ultrathin ZnO film deposited at 25°C was rich in atomic defects, such as oxygen vacancies or impurities [10,11]. The optical and physical properties observed in various ZnO structures have been the subject of extensive researches [10,12-15], and it is generally conceived that the green-yellow PL emission in ZnO is primarily due to oxygen-related defects residing near the surface. In our study, the ultrathin ZnO film deposited on Si nanopillars at 200°C reveals a strong UV emission band at 374 nm (approximately 3.31 eV) with a full width at half maximum approximately equal to 148 meV (see the inset of Figure 4). This UV emission is attributed to the band-edge emission resulting from the recombination of free excitons through the intrinsic energy gap of ZnO [16]. It is true that the bandgap of ZnO is 3.37 eV [17] at room temperature. Free excitons in ZnO, however, have a binding energy of about 60 meV [18], that is to say, the corresponding luminescence is expected at 3.31 eV. In general, the PL emission without band-to-band luminescence at room temperature is expected. In addition, the ZnO/Si nanopillar heterostructures have a much larger surface-to-volume ratio; therefore, an extraordinary surface state-related emission was showed in our sample, as is evident in Figure 4 (solid red circles). Moreover, comparing the UV emission intensity of the ZnO/Si nanopillar heterostructures (solid red circles) and that of the ZnO/Si substrate (solid blue squares), it was found that the optical properties were different for the ultrathin ZnO films deposited on the Si substrate and Si nanopillars at a growth temperature of 200°C. As can be seen from the results, the two spectra indicate identical characteristics of PL emission. The UV intensity of ZnO/Si nanopillars is about five times of magnitude than that of ZnO/Si substrate, which resulted from the large surface-to-volume ratio of the ZnO/Si nanopillar heterostructures [10]. On the basis of the PL measurements, the visible emission was almost suppressed (shown in Figure 4), indicating that the resulting ultrathin ZnO films, including those on Si nanopillars or Si substrate, possess good qualities. The current results, therefore, have demonstrated a viable way of obtaining efficient UV emission from ultrathin ZnO films.

## Conclusions

In summary, we have demonstrated that by the combination of self-assembled Ag nanoislands and the subsequent dry etching process to form a Si nanopillar array, ZnO films were deposited on Si nanopillars by ALD, forming ZnO/Si nanopillar heterostructures. According to such results, it was found that compared to the ZnO/Si substrate, the UV emission intensity was strongly enhanced in the ZnO/Si nanopillar heterostructures, which is mainly ascribed to the large surface-to-volume ratio. It is obvious that compared to most of the techniques employed in obtaining similar structures, the current process of using these self-assembled Ag islands as metal nanomasks with subsequent dry etching can produce Si nanopillars, which also has the advantages of both simplicity and effectiveness.

## Competing interests

The authors declare that they have no competing interests.

## Authors’ contributions

TYC carried out the fabrication of the ZnO/Si nanopillar heterostructure samples and measured their characterization. CLD supervised the work of TYC. Both authors read and approved the final manuscript.

## Authors’ information

Mr. TYC received his MS degree in mechanical engineering from Feng Chia University, Taiwan, in 1999. He is currently a candidate for a doctor's degree at the Department of Mechanical Engineering, National Chung Hsing University and an instructor at the Department of Mechanical Engineering, National Chin-Yi University of Technology, Taiwan. His research interests are nanotechnology and thin film materials.

Dr. CLD received his MS degree in applied mechanics from National Taiwan University, Taiwan, in 1993 and his Ph.D. degree in mechanical engineering from Nation Taiwan University, in 1997. He is currently a professor at the Department of Mechanical Engineering, National Chung Hsing University, Taiwan. His research interests are microsystem technology, nanotechnology, microsensors, and microactuators.
